# High intensity focused ultrasound-induced bubbles stimulate the release of nucleic acid cancer biomarkers

**DOI:** 10.1186/2050-5736-3-S1-O64

**Published:** 2015-06-30

**Authors:** Tatiana Khokhlova, John Chevillet, George Schade, Maria Giraldez, Yak-Nam Wang, Joo Ha Hwang, Muneesh Tewari

**Affiliations:** 1 University of Washington, Seattle, Washington, United States; 2 Institute for Systems Biology, Seattle, Washington, United States; 3 University of Michigan, Ann Arbor, Michigan, United States

## Background/introduction

Prostate biopsy for prostate cancer (PCa) is invasive with associated morbidity and several diagnostic limitations, suggesting the need for a new approach. Recently, several nucleic acid cancer biomarkers (e.g., microRNA and mutant DNA) have been identified and shown promise for improving cancer diagnostics. However, the abundance of these biomarker classes in the circulation is low, impeding reliable detection and adoption into clinical practice. In order to stimulate the release of these intracellular biomarkers, the exposures optimized for mechanical disruption of cells may be desirable.

Here, two approaches based on HIFU-induced bubble activity were tested for their ability to stimulate release of cancer-associated microRNAs in a heterotopic syngeneic rat prostate cancer model. In the first approach, tumor tissue was locally liquefied with boiling histotripsy (BH) - a HIFU technique utilizing millisecond-long pulses to create boiling bubbles via rapid shockwave heating. The interaction of shocks with the ensuing vapor cavity fractionates tissue with negligible thermal effect. In the second approach HIFU-induced inertial cavitation was used for permeabilization of tumor tissue and vasculature.

## Methods

Putative miRNA biomarkers were identified using RT-PCR array profiling of the syngeneic MatLyLu rat PCa cell line. Adult intact male Copenhagen rats were then subcutaneously grafted with the MatLyLu cells. When the tumors were >1cm, the rats were assigned to one of two HIFU treatment groups: HIFU optimized for inertial cavitation activity (focal peak negative pressure 16 MPa, 1 ms pulses, duty factor 0.001, N=6), BH (intensity ~20kW/cm2, 1% duty factor, N=8) or a to a control group (N=6) that received sham treatment. Treatments were performed in a heated water tank using a single-element 1.5 MHz HIFU transducer (45 mm radius of curvature, 64 mm aperture) under ultrasound image guidance. Blood samples were collected immediately prior to treatment and serially over a 24-hour time course. Specimens were immediately processed into plasma and miRNAs extracted. Plasma concentrations of candidate tumor-derived miRNAs were measured via quantitative RT-PCR and compared with ANOVA and the Mann-Whitney test.

## Results and conclusions

Following sham procedure, no significant changes were observed in the relative plasma concentrations of any evaluated miRNA. Conversely, following both cavitation-based and BH treatments, the relative plasma concentrations of the putative PCa-associated miRNAs miR-34c and miR-196a increased significantly while the relative concentration of the broadly expressed, non-PCa specific miR-16 was not significantly altered. PCa-associated miRNA concentrations peaked at 0.25 hr (10-23-fold) from initiation of HIFU treatment, remained significantly elevated for 3 hours, and then returned to baseline within 24 hours. Histologic examination of excised tumor confirmed complete fractionation of targeted tumor by BH and localized areas of intraparenchymal hemorrhage and tissue disruption by cavitation-based treatment. These data suggest a clinically useful application of HIFU-induced bubbles for non-invasive molecular biopsy.

**Figure 1:**
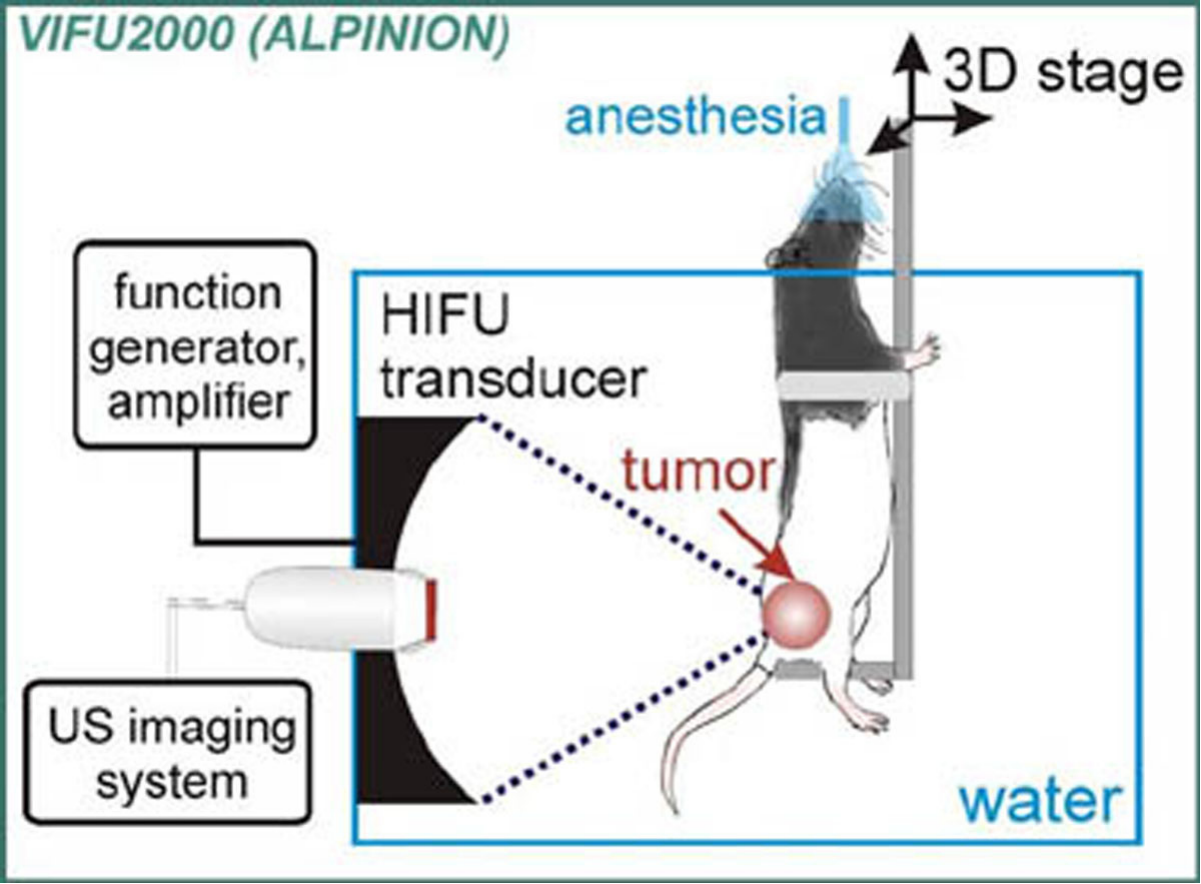
Experimental setup

**Figure 2:**
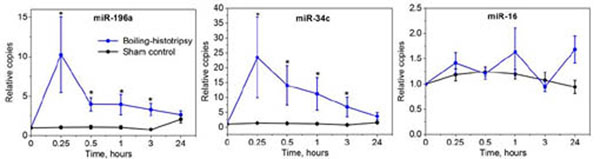
Changes in relative plasma concentration (copies) of broadly expressed miRNA miR-16 and tumor-associated miRNAs miR-34c and miR-196a following boiling-histotripsy (n=8) or sham treatment (n=6) of subcutaneous rat prostate cancer tumors (*p<0.05)

